# *De novo* transcriptome sequencing and gene expression profiling with/without B-chromosome plants of *Lilium amabile*

**DOI:** 10.5808/GI.2019.17.3.e27

**Published:** 2019-09-16

**Authors:** Doori Park, Jong-Hwa Kim, Nam-Soo Kim

**Affiliations:** 1 Department of Molecular Biosciences, Kangwon National University, Chuncheon 24341, Korea; 2 Department of Horticulture, Kangwon National University, Chuncheon 24341, Korea; 3 Oriental Bio-herb Research Institute, Kangwon National University, Chuncheon 24341, Korea; 4 Institute of Bioscience and Biotechnology, Kangwon National University, Chuncheon 24341, Korea

**Keywords:** A chromosomes, B chromosomes, differentially expressed genes, *Lilium amabile*, transcriptome

## Abstract

Supernumerary B chromosomes were found in *Lilium amabile* (2n = 2x = 24), an endemic Korean lily that grows in the wild throughout the Korean Peninsula. The extra B chromosomes do not affect the host-plant morphology; therefore, whole transcriptome analysis was performed in 0B and 1B plants to identify differentially expressed genes. A total of 154,810 transcripts were obtained from over 10 Gbp data by *de novo* assembly. By mapping the raw reads to the *de novo* transcripts, we identified 7,852 differentially expressed genes (log_2_FC > |10|), in which 4,059 and 3,794 were up-and down-regulated, respectively, in 1B plants compared to 0B plants. Functional enrichment analysis revealed that various differentially expressed genes were involved in cellular processes including the cell cycle, chromosome breakage and repair, and microtubule formation; all of which may be related to the occurrence and maintenance of B chromosomes. Our data provide insight into transcriptomic changes and evolution of plant B chromosomes and deliver an informative database for future study of B chromosome transcriptomes in the Korean lily.

## Introduction

B chromosomes are supernumerary chromosomes that are present in addition to the standard chromosome complements in eukaryotes. Although they are not essential for normal growth and development of an organism, they have been identified in approximately 15% of all eukaryotes, including numerous plant and animal species [1,2]. B chromosomes differ from A chromosomes with distinctive features such as lack of chromosome pairing during meiosis and non-Mendelian inheritance. They may be present and variable in number in some individuals of a population. Individuals with B chromosomes usually do not display obvious phenotypic differences from normal diploids, and B chromosomes do not offer selective advantage to the host in most cases [1-3]. However, supernumerary chromosomes can be detrimental to the host, as observed in rye which more than 8 B chromosomes results in low fertility [4].

Although the mechanism of *de novo* B chromosome occurrence is unclear, recent studies have delineated the origin of B chromosomes in several species. Next-generation sequencing of A and B chromosomes sorted from rye (*Secale cereale*) revealed that the B chromosomes were a mosaic of host genome and organellar DNA sequences [5]. Integrated genomic analyses after B chromosome microdissection showed that B chromosomes contain thousands of sequences derived from A chromosomes of the entire ancestral karyotype of the cichlid fish (*Astatotilapia latifasciata*) [6]. Because B chromosomes exhibit no obvious phenotypic effects and are selectively neutral, B chromosomes were often regarded as “selfish” chromosomes, containing highly repetitive heterochromatic elements without functional genes [3]. Indeed, sequences of maize B chromosomes were enriched with highly repetitive DNA sequences that showed no homology with known genes other than retrotransposon sequences and miniature inverted-repeat transposable elements [7]. However, these repeat sequences are not specific to B chromosomes because they are also present on A chromosomes. In cichlid fish, B chromosomes were shown to be filled with a variety of transposable elements and simple repeats, with low complexity sequences constituting only a small portion of the genetic material. Nonetheless, high integrity genes involved in cell division were detected in the cichlid fish B chromosomes [6]. Moreover, the idea of transcriptional inertness of B chromosome genes was declined because of the recent discovery of several transcriptionally active genes in B chromosomes [8,9]. For instance, ribosomal RNA genes of the B chromosomes were actively transcribed in the grasshopper (*Eyprepocnemis plorans*) [10,11]. Huang et al. [9] conducted transcriptome analyses in 1B and 0B maize plants, in which several genes were localized to B chromosomes, and three of the genes encoded long terminal repeat (LTR)–retrotransposons. These genes have A chromosome paralogs, and expression of B chromosome paralogs was confirmed by bioinformatic analyses. Moreover, whole transcriptome profiles were altered by the presence of B chromosomes, displaying advancement with increased B chromosome number [9]. In *Drosophila melanogaster*, presence of B chromosomes may act as an enhancer or suppressor of position-effect variegation, depending on the genetic background [12]. Lin et al. [13] analyzed transcript profiles from inbred maize strains by cDNA-AFLP (amplified fragment length polymorphism) and found that B chromosomes contained transcriptionally active genes and altered A chromosome gene transcription compared to the maize strain lacking B chromosomes.

The genus *Lilium* includes about 110–115 species that are widely distributed in temperate zones of the Northern Hemisphere [14]. Northeast Asia is the proposed center of origin for the *Lilium* genus, and 13 *Lilium* species have been identified in the Korean Peninsula [14,15]. Since the first report on B chromosomes in *Lilium japonicum* in 1932 [16], 33 *Lilium* species have been identified to carry B chromosomes (http://www.bchrom.csic.es/), which accounts for approximately 30% of the species in the *Lilium* genus. *Lilium amabile* (2n = 2x = 24) is an endemic lily that grows wild throughout the Korean Peninsula. The lilies are easily found on hills and small mountains, but not high mountains and deep forest. *L. amabile* contains four types of B chromosomes: one large, almost acrocentric; one large, acrocentric that differs in arm index from other large B chromosomes; one small, acrocentric; and one small, metacentric chromosome. There are 12 cytotypes of *L. amabile*, depending on B chromosome composition [17]. Currently, there are no reports on gene expression in *L. amabile*; our study contains *de novo* transcriptome profiles of the 1 B and 0B chromosomes of this plant species.

## Methods

### Plant materials and RNA extraction

**Fig. 1.**
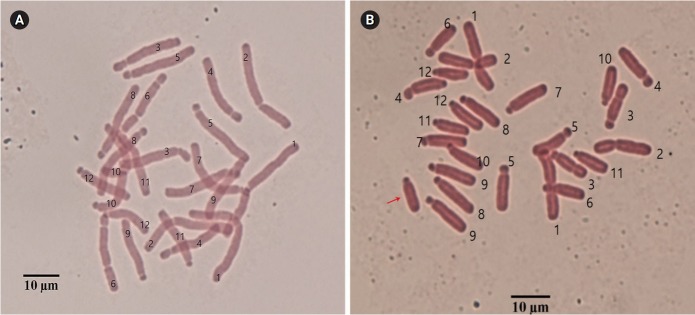
Somatic chromosome complements of 0B (A) and 1B (B) chromosomes from *Lilium amabile*. Chromosome numbers are indicated, and the arrow indicates the supernumerary B chromosome.

Before extraction of mRNA, chromosome numbers were confirmed from root-tip cells. We selected three plants with long acrocentric B chromosomes (Fig. 1). Root-tip collection and chromosome preparation were followed as previously described by Nguyen et al. [18].

Plants were grown in 5-inch wide by 5-inch deep pots with 10 h dark (20℃) and 14 h light (25℃) cycles. For RNA sequencing, 300 mg of fully expanded leaf tissues were collected from each of the three independent biological replicates for 0B and 1B chromosome–containing plants, respectively. All samples were immediately frozen in liquid nitrogen and then used to extract RNA. Three biological replicates for each sample were mixed together to reduce inter-individual variations. Total RNA was isolated using QIAGEN plant RNA extraction kit according to the manufacturer’s instructions (Qiagen, Hilden, Germany). The quality and concentration of RNA were assessed using the Agilent 2100 Bioanalyzer (Agilent Technologies, Santa Clara, CA, USA) and Nanodrop 2000 spectrophotometer (Thermo Fisher Scientific, Waltham, MA, USA) with parameters RIN ≥ 7, 28S:18S > 1, OD260/280 ≥ 2.

### Sequencing and library construction

cDNA libraries from each sample were prepared using Truseq mRNA library prep kit (Illumina, San Diego, CA, USA) with a library size of 500 bp. Samples were sequenced on an Illumina Hiseq3000 with paired-end method. Raw data were deposited in the NCBI Short Read Archive (SRA) under the following accession numbers: SRR8316493 and SRR8316494.

### Transcriptome assembly, annotation, and functional analysis

After filtered raw data, we obtained clean and qualified reads (Phred quality score > 20, length > 50 bp) and the filtered clean reads were used to perform *de novo* assembly using Trinity program (https://github.com/trinityrnaseq). The assembled transcripts were then annotated through BLAST analysis against the NCBI nucleotide database (e-value of 1E+03). For functional annotation, we mapped transcripts onto Gene Ontology (GO) and Kyoto Encyclopedia of Genes and Genomes (KEGG) databases.

### Quantification of differentially expressed gene

For quantitative real-time polymerase chain reaction (qRT-PCR), RNA was extracted from leaves of the same plants used in RNA sequencing (RNA-seq) analysis. Total RNA was isolated using the same method as described for RNA sequencing. Gene expression was calculated using FPKM (fragments per kilobase of transcripts per million mapped reads). All transcripts were used to calculate its expression level and coverage. The differences in gene expression between 0B and 1B chromosome plant samples were assessed statistically by p-values; additionally, the false discovery rate (FDR) was used to determine the parameter for classification of significant differentially expressed genes (DEGs) (FDR < 0.05). Expression values were represented by log2 ratio.

### Quantitative real-time polymerase chain reaction

qRT-PCR was performed to verify the expression value of nine DEGs. For data normalization, *GAPDH* gene expression was used as an internal reference. All samples were individually analyzed for three biological replicates. PCR was conducted using SYBR Green fluorescent dye in a 7500 Real-Time PCR System (Applied Biosystems, Foster City, CA, USA). Reaction mixture was prepared using Thunderbird SYBR qPCR Mix (TOYOBO, Japan) following the manufacturer’s instructions. The cycling conditions were as follows: 95℃ for 1 min, 40 cycles of 95℃ for 15 s, 60℃ for 1 min. Melting curves for PCR products were analyzed under the following conditions: 95℃ for 15 s, cooling to 60℃ for 1 min, and then gradual heating at 0.1℃/s to a final temperature of 95℃. The qRT-PCR data were analyzed using 2^-ΔΔCt^ method. Mean and standard deviations were calculated with triplicate data from three independent biological replicates. Primer information used in this study was described in Supplementary Table 1.

## Results

### Sequencing and *de novo* assembly of the transcriptomes

Sequencing summaries and *de novo* assembly of the transcriptome results are presented in Supplementary Table 2–4. We obtained over 10 Gbp raw data from leaf samples of 0B and 1B plants. For *de novo* sequence assembly, quality trimming of raw reads for 83.76% in 0B and 81.69% in 1B plant samples resulted in 77,087 genes and 154,810 transcripts with an average length 791 bp. It was estimated that approximately 99% of the reads were mapped to *de novo* transcripts. All sequence reads have been deposited in NCBI Sequence Read Archive (https://www.ncbi.nlm.nih.gov/sra). The BioProject and SRA accessions are PRJNA509487, SRR8316493, and SRR8316494, respectively.

### Differential expression profiling of significant GO terms

**Fig. 2.**
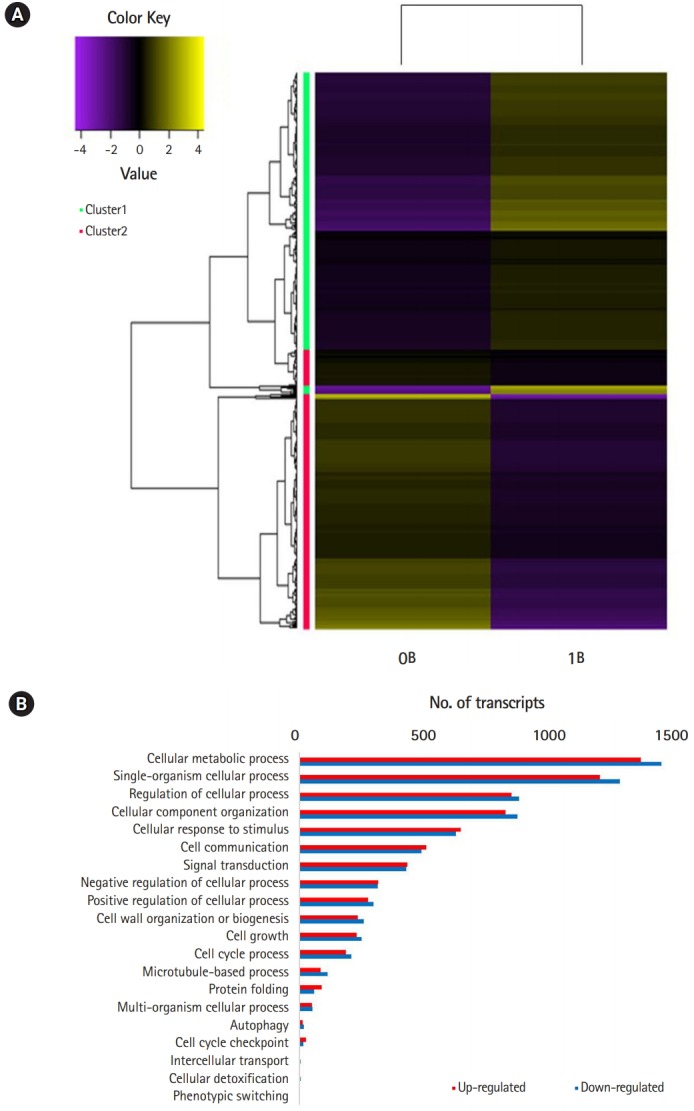
(A) Heat map of the differentially expressed genes between 0B and 1B *Lilium amabile* plants. (B) Gene Ontology enrichment analysis of differentially expressed gene.

Of the 154,810 transcripts, 4,157 up-and 3,891 down-regulated genes were found in B-chromosome–containing plants (FDR < 0.05). At the threshold of differential expression value log_2_FC > |2|, 4,059 transcripts were down-regulated and 3,794 transcripts were up-regulated, accounting for 5.1% of the total transcripts (Table 1). At the stringent threshold log_2_FC > |10|, we obtained 552 up- and 490 down-regulated transcripts, respectively, accounting for 0.6% of the total transcripts. Heat map analysis revealed differentially expressed patterns that were grouped into two clusters using K-mean clustering (Fig. 2A).

**Fig. 3.**
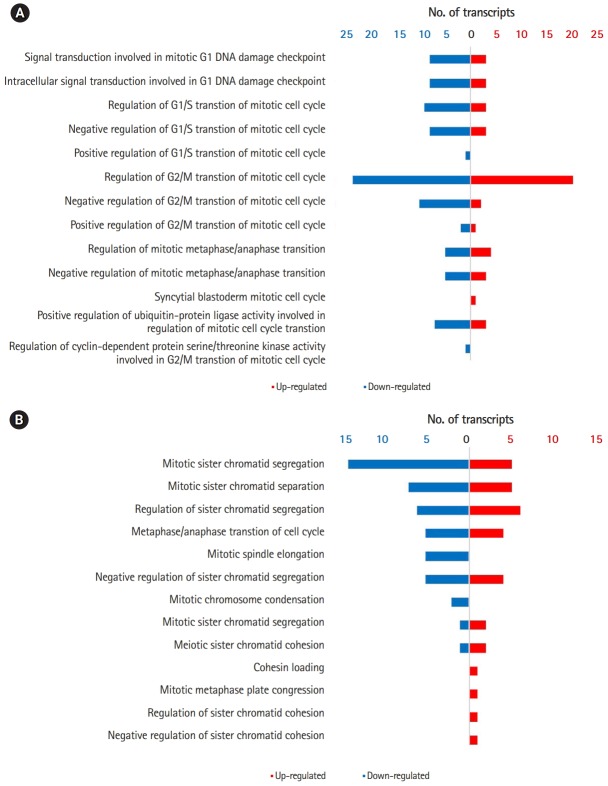
(A) Differentially expressed cell cycle–related genes in 1B plants of *Lilium amabile*. Red and blue bars indicate up-and down-regulated differentially expressed gene (DEG) in 1B plants, respectively. (B) Differentially expressed chromosome segregation related genes in *L. amabile*. Red and blue bars indicate up-and down-regulated DEG in 1B plants, respectively.

Comparison of GO terms between 0B and 1B chromosome plants revealed multiple DEGs in cellular processes that could potentially account for the *de novo* occurrence and segregation of B chromosomes (Fig. 2B) [6,7]. Fig. 3 show significantly enriched GO terms involved in the cell cycle and chromosome segregation of genes that were down-regulated in the presence of B chromosomes compared to normal karyotype plants. We found 38 differentially regulated cell cycle–related genes, of which 20 were up-regulated and 18 were down-regulated (Supplementary Tables 5 and 6). Of the 68 genes related to chromosome segregation, 41 were up-regulated and 22 were down-regulated (Supplementary Table 6). The overall DEGs listed in significant GO terms are listed in Supplementary Table 7.

### qRT-PCR validation of DEG profiles

**Fig. 4.**
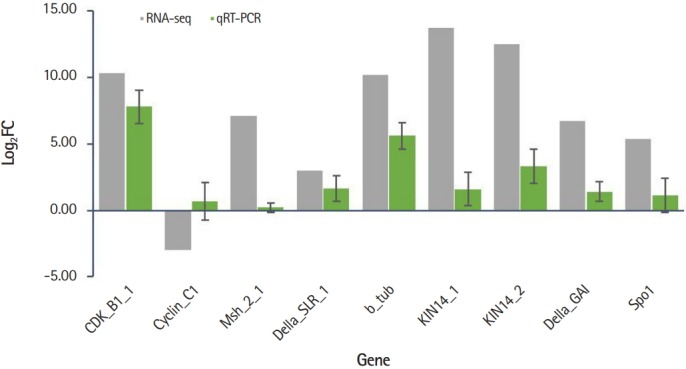
Quantitative real-time polymerase chain reaction (qRT-PCR) validation of differentially expressed gene involved in cell cycle and chromosome segregation. X-axis represents gene symbols and Y-axis shows relative gene expression values presented by log_2_FC.

The expression profiles of nine genes involved in the cell cycle (*CDKB1* and *CyclinC1*) and chromosomal segregation (*Msh, Della, b-tubulin, KIN*, and *SPO*) were validated by qRT-PCR. Expression values were compared by calculating log_2_FC obtained by the RPKM values from RNA-seq data and Ct values from qRT-PCR. Of the nine genes, eight genes were consistent with RNA-seq and qRT-PCR results and one gene (*Cyclin C1*) showed opposite expression patterns in RNA-seq vs. qRT-PCR (Fig. 4). The raw expression data showed that there were two isoforms of *Cyclin C1*, one was up-expressed whereas another was down-expressed. Thus, the obscure result of *Cyclin C1* of RNA-seq versus qRT-PCR may require further investigation. Our results indicate that despite the limited number of DEG analyzed in the presence or absence of B chromosomes, genes related to the cell cycle and chromosome segregation were affected by the presence of B chromosomes.

## Discussion

B chromosomes are supernumerary chromosomes in eukaryotes. The current report contains differential transcriptome profiles between 0B and 1B chromosome plants of *L. amabile*, an endemic lily in Korea. Transcriptome profiles were reported from the *Lilium* genus [19-22], but not from the *L. amabile*. Therefore, *de novo* assembly was conducted with *L. amabile* transcriptomes derived from fully expanded leaves. Our results are comparable to profiles from other *Lilium* species. Villacorta-Martin et al. [19] reported 42,430 genes from 121,572 transcripts that were derived from bulbs of commercial cultivar of *L. longiflorum*. Hu et al. [23] also reported transcriptomes from petals of cultivars from Sorbonne and Novano to elucidate differences in floral scent, of which 124,233 NCBI UniGene clusters from 229,128 transcripts were reported. Thus, our volume of data from 154,810 transcripts of 77,087 genes falls between these two studies.

In our study, approximately 20% of the transcripts from leaves were differently expressed between the 0B and 1B chromosome plants at the differential expression value of log_2_FC > |2|. However, only 0.6% of the total transcripts were differentially expressed in the stringent threshold of log_2_FC > |10| (FDR < 0.05). Our results are similar to the report of rye B chromosome analysis [24], in which approximately 0.6% cDNA-AFLP analysis showed differences between 0B and 1B chromosome plants, and there were 16 putative B chromosome-associated transcripts. Chromosome imbalance usually exhibits deleterious phenotypic consequences in A chromosome aneuploids; however, B chromosomes do not affect phenotype unless attaining certain numbers [4,25]. Disrupted genetic homeostasis by aneuploidy often impacts global modulation of gene expression, which is often obvious in complex ways [26]. Plants with a single B chromosome do not reveal morphological phenotypic differences from normal diploid plants in our study. Thus, further analyses, including gene expression profiles in multiple B chromosome plants, may provide explanation for the morphological indifferences. Presence of B chromosomes can disturb the expression of genes in A chromosomes. In maize, transcription of A chromosome genes was altered in the presence of B chromosomes, and the extent of alteration coincided with the number of B chromosomes [9]. Because most genetic material of B chromosomes is derived from A chromosomes [5,6], genes in B chromosomes can be paralogous to genes in A chromosomes. Most of the paralogous genes in B chromosomes become pseudogenes and are transcriptionally silenced because B chromosomes are selectively neutral [25-27]. Nevertheless, extra gene copy transcription from B chromosomes can result in knock-down mRNA of the paralogous genes of the A chromosome by RNA-directed RNA polymerase–induced RNAi degradation [8]. The proteins originated from B chromosomes can also act as an enhancer or suppressor of A chromosome genes [12]. Alternately, aberrant gene expression copies may be equalized by gene dosage compensation [8]. In our analysis, it was not possible to map the transcripts on the chromosomes because the gene map or chromosome substitution are not available for *Lilium*. Also, low-copy gene detection by fluorescent in situ hybridization is not a readily available technique for *Lilium* due to the huge genome size (36.5 pg) [28]. Nonetheless, the differentially expressed transcripts may mostly be products of genes on A chromosomes, but some of them may reside on the extra B chromosomes.

Our results showed that the DEG included mostly genes involved in the cell cycle and chromosome segregation [29,30]. Cyclin-dependent cycle (*CDC*), cyclin-dependent kinase (*CDK*), and cyclin (*CYC*) genes were included in the DEG related to the cell cycle. CDC proteins are cell cycle controllers that associate with cyclins depending on developmental and environmental cues [29]. Control of chromosome segregation is essential for chromosome ploidy and stability in both somatic and meiotic cells [30]. Kinesins are motor proteins that move along microtubule filaments and are critically involved in chromosome segregation during mitosis and meiosis [30]. Interestingly, we found 31 *KIN* genes that were differentially expressed in our study. Of these genes, 23 were up-regulated and eight were down-regulated in 1B chromosome plants. Chromosome pairing maintains homologous chromosomes until anaphase to ensure proper chromosome segregation [30]. Several genes involved in chromosome pairing were also included in the DEG from our analyses [31]. We cannot infer the precise mechanism of DEG for *de novo* B chromosome occurrence and transmission at this stage. Reverse genetic approaches remain a challenge but may provide answers to *de novo* occurrence of B chromosomes for future studies.

In conclusion, B chromosomes are supernumerary chromosomes present in eukaryotes; however, the presence of these additional chromosomes does not display obvious phenotypic effects. B chromosomes are found in numerous species in the *Lilium* genus. Prior to the current report, no molecular or genomic studies were conducted to characterize B chromosomes in the *Lilium* species. In this study, we performed RNA-seq analysis from leaf tissues of 0B and 1B chromosome *L. amabile* plants. Of the 154,810 transcripts detected, 552 were up-regulated and 490 were down-regulated in 1B plants compared to the 0B plants with the differential expression value of log_2_FC > |10|. Most of the DEG included cell cycle and chromosome segregation related genes, which may be associated with the *de novo* occurrence and maintenance of B chromosomes. The DEG from the current analysis will provide a valuable resource for studying the accumulation of B chromosomes in plants and plant evolution.

## Figures and Tables

**Table 1.** Number of up- and down-regulated genes in 1B chromosome plant

	Total	log_2_FC > |2|	log_2_FC > |10|
Up-regulated	15794	11644	552
Up (FDR < 0.05)	4157	4059	552
Down-regulated	16513	12320	490
Down (FDR < 0.05)	3891	3794	490

FDR, false discovery rate.

## Supplementary Materials

Supplementary Table 1. Primer sequences for qRT-PCR analysis

Supplementary Table 2. Sequencing summary

Supplementary Table 3. *De novo* assembly summary

Supplementary Table 4. PReads alignments against *de novo* transcripts

Supplementary Table 5. DE profiles of cell cycle related genes in *Lilium amabile*

Supplementary Table 6. DE profiles of chromosome segregation related genes in *Lilium amabile*

Supplementary Table 7. Lists of differentially expressed genes in 1B plants
