# Supplementary material for: De novo transcriptome sequencing and gene expression profiling with/without B-chromosome plants of Lilium amabile
Source: Genomics Inform. 2019 Sep 16;17(3):e27. doi: 10.5808/GI.2019.17.3.e27 (PMC6808634; doi:10.5808/GI.2019.17.3.e27)
Supplement: Supplementary Table 1. — Primer sequences for qRT-PCR analysis [file gi-2019-17-3-e27-suppl1.pdf]

Supplementary Table 1. Primer sequences for qRT-PCR analysis

No.	Primer name	Left primer	Right primer
1	CDK_B1_1	GCTGGCATGAATACGAGCAA	TCAAGGCTGTCGAAGTAGGG
2	Cyclin_C1	CTCTCCTCTGCTTTTGACGC	TTGGAAGCTGGCTCAACATG
3	Msh2_1	TTTCACGGGAATTGGGAAAC	CTGCAACACCTGGCAACATT
4	DELLA_SLR1	TCTCCCATCCCATCTTTGCT	CGCTGCTTTCCTGGTTCTCT
5	b-tubulin	TTTGGGAGTGTCTGGTGTGC	ACTTTCGAAAAGGGCGACAA
6	KIN_14_1	ACTGGAAAGGTCGACGGTGG	CTTGTCGAAGCTGGCTGTGC
7	KIN_14_2	ACTGCATCATCCCTTCCCCG	ACTGAGGCTTGCACACCACT
8	DELLA_GAI	CATTACAACCCCACCGACCT	TAGAGCTGATCCGTCCACGA
9	Spo1	CGGAGATGTATCGGTGCTTT	CCTTCCCAATCTCCTCTTCC

qRT-PCR, quantitative real-time polymerase chain reaction.
