# Supplementary material for: De novo transcriptome sequencing and gene expression profiling with/without B-chromosome plants of Lilium amabile
Source: Genomics Inform. 2019 Sep 16;17(3):e27. doi: 10.5808/GI.2019.17.3.e27 (PMC6808634; doi:10.5808/GI.2019.17.3.e27)
Supplement: Supplementary Table 2. — Sequencing summary [file gi-2019-17-3-e27-suppl2.pdf]

Supplementary Table 2. Sequencing summary

Sequencing data	0B	1B
Raw data		
No. of reads	66,884,332	70,788,632
Read length (bp)	10,099,534,132	10,689,083,432
Trimmed data		
No. of reads	58,556,960	60,635,380
Read length (bp)	8,459,699,995	8,732,208,616
Trimmed percentage (%)	83.76	81.69
Corrected data		
No. of paired reads	56,311,234	58,393,764
Paired reads length (bp)	7,972,672,604	8,230,577,997
No. of single reads	545,598	606,185
Single reads length (bp)	57,961,048	64,347,261
