# Supplementary material for: De novo transcriptome sequencing and gene expression profiling with/without B-chromosome plants of Lilium amabile
Source: Genomics Inform. 2019 Sep 16;17(3):e27. doi: 10.5808/GI.2019.17.3.e27 (PMC6808634; doi:10.5808/GI.2019.17.3.e27)
Supplement: Supplementary Table 3. — De novo assembly summary [file gi-2019-17-3-e27-suppl3.pdf]

**Supplementary Table 3.** *De novo* assembly summary

| | Gene | Transcript |
|---|---|---|
| Total number | 77,087 | 154,810 |
| Total length (bp) | - | 112,403,282 |
| Average length (bp) | - | 791 |
