# Supplementary material for: De novo transcriptome sequencing and gene expression profiling with/without B-chromosome plants of Lilium amabile
Source: Genomics Inform. 2019 Sep 16;17(3):e27. doi: 10.5808/GI.2019.17.3.e27 (PMC6808634; doi:10.5808/GI.2019.17.3.e27)
Supplement: Supplementary Table 4. — PReads alignments against de novo transcripts [file gi-2019-17-3-e27-suppl4.pdf]

Supplementary Table 4. Reads alignments against *de novo* transcripts

Sample	Total No. of reads used	Mapped reads	
		No. of reads	Mapping percentage (%)
2n = 24	56,311,234	55,801,289	99.09
2n = 24 + 1B	58,393,764	57,612,487	98.66
