# Supplementary material for: De novo transcriptome sequencing and gene expression profiling with/without B-chromosome plants of Lilium amabile
Source: Genomics Inform. 2019 Sep 16;17(3):e27. doi: 10.5808/GI.2019.17.3.e27 (PMC6808634; doi:10.5808/GI.2019.17.3.e27)
Supplement: Supplementary Table 5. — DE profiles of cell cycle related genes in Lilium amabile [file gi-2019-17-3-e27-suppl5.pdf]

Supplementary Table 5. Differentially expressed profiles of cell cycle related genes in *Lilium amabile*

Gene symbol	Annotation	Total	Up -regulated	Down -regulated
CDC	Cell division cycle protein 27 homolog B isoform X1	2	1	1
CDC	Cell division cycle protein 48 homolog	7	3	4
CDC	Cell division protein FtsZ homolog 1, chloroplastic-like	1	1	-
CDKB;11	Cyclin-dependent kinase 11B-like	1	-	1
CDKB1;1	Cyclin-dependent kinase B1-1	2	2	-
CDKC;2	Cyclin-dependent kinase C-2-like	1	-	1
CDKD;1	Cyclin-dependent kinase D-1	1	1	-
CDKF;1	Cyclin-dependent kinase F-1-like	2	1	1
CDKG;2	Cyclin-dependent kinase G-2-like	2	1	1
CDKi1C	Cyclin-dependent kinase inhibitor 1C-like	1	1	-
CDKi4	Cyclin-dependent kinase inhibitor 4-like	2	1	1
CDKi5	Cyclin-dependent kinase inhibitor 5-like	1	-	1
CDT1	CDT1-like protein a, chloroplastic	1	-	1
CYC;SDS	Cyclin-SDS-like isoform X2	1	1	-
CYCA;1	Cyclin-A1-1-like	1	-	1
CYCA;2;1	Cyclin-A2-1-like	1	1	-
CYCB;1;2	Cyclin-B1-2-like	2	1	1
CYCC;1;1	Cyclin-C1-1-like isoform X1	2	1	1
CYCP3;1	Cyclin-P3-1-like	1	-	1
E2FB	Transcription factor E2FB-like	4	2	2
FBL	BTB/POZ domain-containing protein FBL11 isoform X2	1	1	-
ORC	Origin of replication complex subunit 1 isoform X1	1	1	-
