# Supplementary material for: De novo transcriptome sequencing and gene expression profiling with/without B-chromosome plants of Lilium amabile
Source: Genomics Inform. 2019 Sep 16;17(3):e27. doi: 10.5808/GI.2019.17.3.e27 (PMC6808634; doi:10.5808/GI.2019.17.3.e27)
Supplement: Supplementary Table 6. — DE profiles of chromosome segregation related genes in Lilium amabile [file gi-2019-17-3-e27-suppl6.pdf]

Supplementary Table 6. Differentially expressed profiles of chromosome segregation related genes in *Lilium amabile*

Gene symbol	Annotation	Total	Up -regulated	Down -regulated
APC	Anaphase-promoting complex	6	2	4
Mus81	Crossover junction endonuclease MUS81 isoform X2	1	-	1
CULLIN	Cullin-1 isoform X1	1	1	-
MLH1	DNA mismatch repair protein MLH1	2	1	1
MSH1	DNA mismatch repair protein MSH1, mitochondrial	1	1	-
MSH2	DNA mismatch repair protein MSH2	3	2	1
PMS1	DNA mismatch repair protein PMS1 isoform X1	1	1	-
XRCC4	DNA repair protein XRCC4	1	1	-
HSP5	Heat shock 70 kDa protein 5-like	1	1	-
KINs	Kinesin family protein	31	23	8
BUBR1	Mitotic spindle checkpoint protein BUBR1	2	2	-
RTEL1	Regulator of telomere elongation helicase 1 isoform X2	2	0	2
AURK	Serine/threonine-protein kinase Aurora-1	1	1	-
PDS5	Sister chromatid cohesion protein PDS5 homolog A isoform X2	1	-	1
Skp1	SKP1-interacting partner 15-like, partial	1	-	1
SMC	Structural maintenance of chromosomes protein 1	1	1	-
ZIP	TPR repeat-containing protein ZIP4	2	2	-
TUBB	Tubulin beta 2	3	1	2
TUBB1	Tubulin beta-1 chain-like, partial	1	1	0
TUBG	Tubulin gamma-2 chain	1	0	1
