# Supplementary material for: De novo transcriptome sequencing and gene expression profiling with/without B-chromosome plants of Lilium amabile
Source: Genomics Inform. 2019 Sep 16;17(3):e27. doi: 10.5808/GI.2019.17.3.e27 (PMC6808634; doi:10.5808/GI.2019.17.3.e27)
Supplement: Supplementary Table 7. — Lists of differentially expressed genes in 1B plants [file gi-2019-17-3-e27-suppl7.pdf]

Supplementary Table 7. Lists of differentially expressed genes in 1B plants

Gene classification	Gene symbols	Annotation	Up-regulated (RPKM)	Down-regulated (RPKM)
Cell cycle	*Cyclins*	PREDICTED: cyclin-A2-1-like	10.17	-
		cyclin-SDS-like isoform X2	8.28	-
		cyclin-C1-1-like isoform X1	5.66	-
		cyclin-B1-2-like	4.45	-
		cyclin-C1-1-like isoform X1	-	–11.59
		PREDICTED: cyclin-A2-1-like	-	–10.58
		PREDICTED: cyclin-A1-1-like	-	–7.74
		PREDICTED: cyclin-P3-1-like	-	–6.58
		Calcyclin-binding protein-like	-	–2.55
		Cyclin-B1-2-like	-	–2.03
	CDK	PREDICTED: cyclin-dependent kinase F-1-like	11.80	-
		PREDICTED: cyclin-dependent kinase B1-1	10.94	-
		PREDICTED: cyclin-dependent kinase B1-1	9.70	-
		Cyclin-dependent kinase G-2-like	7.04	-
		Cyclin-dependent kinases regulatory subunit 1	6.56	-
		PREDICTED: cyclin-dependent kinase D-1	2.51	-

	PREDICTED: cyclin-dependent kinase F-1-like	-	–11.54
	Cyclin-dependent kinase G-2-like	-	–7.84
	PREDICTED: cyclin-dependent kinase 11B-like	-	–6.58
	Cyclin-dependent kinase C-2-like	-	–2.62
CDKI	PREDICTED: cyclin-dependent kinase inhibitor 1C-like	9.38	-
	PREDICTED: cyclin-dependent kinase inhibitor 4-like	8.73	-
	PREDICTED: cyclin-dependent kinase inhibitor 4-like	-	–8.15
	Cyclin-dependent kinase inhibitor 5-like	-	–2.31
CDC	PREDICTED: cell division cycle protein 27 homolog B isoform X1	12.70497868	-
	PREDICTED: cell division control protein 48 homolog B isoform X2	9.685247408	-
	PREDICTED: cell division cycle protein 48 homolog	8.38715009	-
	PREDICTED: cell division cycle protein 48 homolog	7.296185756	-
	PREDICTED: cell division protein FtsZ homolog 1, chloroplastic-like	4.685639108	-
	PREDICTED: cell division cycle protein 27 homolog B isoform X1	-	–12.25621865
	PREDICTED: cell division control protein 48 homolog C-like	-	–11.76020376
	PREDICTED: cell division control protein 48 homolog B isoform X2	-	–10.19555228
	PREDICTED: cell division control protein 48 homolog C	-	–8.337172896
	PREDICTED: cell division cycle protein 48 homolog	-	–2.819047429
	Cell division cycle and apoptosis regulator protein 1-like	-	–1.880654569

	E2F	PREDICTED: transcription factor E2FB-like [Phoenix dactylifera]	9.384993909	-
		PREDICTED: transcription factor E2FB-like [Phoenix dactylifera]	2.315735311	-
		PREDICTED: transcription factor E2FB-like [Phoenix dactylifera]	-	–9.352401165
		PREDICTED: transcription factor E2FB-like [Phoenix dactylifera]	-	–7.839446651
Chromosome segregation	TUBB	PREDICTED: tubulin beta-2 chain-like	10.23	-
		PREDICTED: tubulin beta-1 chain-like, partial	6.74	-
		Beta tubulin 2	-	–8.11
		Beta tubulin 2	-	–7.94
	BUBR	PREDICTED: mitotic spindle checkpoint protein BUBR1	12.00	-
		PREDICTED: mitotic spindle checkpoint protein BUBR1	10.62	-
	KIF	PREDICTED: kinesin-like protein KIN-14R	13.71	-
		Kinesin-like protein KIN-14J isoform X4	12.46	-
		PREDICTED: kinesin-like protein KIN-7L	11.76	-
		PREDICTED: kinesin-like protein KIN-10C	10.99	-
		PREDICTED: kinesin-like protein KIN-12G	10.76	-
		kinesin-like protein KIN-14J isoform X4	9.67	-
		PREDICTED: kinesin-2	9.24	-
		PREDICTED: kinesin-like protein KIN-8A isoform X2	9.09	-
		PREDICTED: kinesin-2	8.39	-

PREDICTED: kinesin-like protein KIN-12E isoform X1	7.94	-
Kinesin-like protein KIN-4C	7.79	-
PREDICTED: kinesin heavy chain	7.12	-
PREDICTED: kinesin-like protein KIN-4A isoform X3	6.74	-
PREDICTED: kinesin-like protein KIN12B	6.74	-
PREDICTED: kinesin-like calmodulin-binding protein homolog isoform X2	6.62	-
PREDICTED: kinesin-like protein KIN-14Q	6.18	-
PREDICTED: kinesin-like protein KIN-7I isoform X1	5.29	-
PREDICTED: kinesin-like protein KIN-12G	5.04	-
kinesin-like protein KIN-4C isoform X2	4.91	-
PREDICTED: kinesin-like protein KIN-7I	3.12	-
PREDICTED: kinesin-like protein KIN-7I isoform X1	2.78	-
PREDICTED: kinesin-like protein KIN-8A isoform X2	2.42	-
PREDICTED: kinesin-like protein KIN-12E isoform X2	2.18	-
Kinesin-like protein KIN-14F	-	–12.11
PREDICTED: kinesin-like protein KIN-12B	-	–8.30
PREDICTED: kinesin-like protein KIN12B	-	–8.27
PREDICTED: kinesin-like protein KIN-12B	-	–8.27
PREDICTED: kinesin-like protein KIN-7E, chloroplastic isoform X2	-	–7.68

		PREDICTED: kinesin-like protein KIN-4A isoform X4	-	–7.57
		PREDICTED: kinesin-like protein KIN-7C, mitochondrial	-	–7.16
		PREDICTED: kinesin-like protein KIN-12B	-	–6.80
	RTEL1	PREDICTED: regulator of telomere elongation helicase 1 isoform X2	-	–6.58
		PREDICTED: regulator of telomere elongation helicase 1 isoform X2	-	–6.58
	AURK	Serine/threonine-protein kinase Aurora-1	2.27	-
	XRCC	PREDICTED: DNA repair protein XRCC4	-	–9.40
synaptonemal complex (SCs)	Others	PREDICTED: DNA mismatch repair protein MLH1	9.81	-
		PREDICTED: DNA mismatch repair protein MLH1	-	–9.62
		Heat shock 70 kDa protein 5-like	-	–7.07
mismatch repair protein	MLH	PREDICTED: DNA mismatch repair protein MLH1	-	–9.62
		PREDICTED: DNA mismatch repair protein MLH1	9.81	-
	MSH	PREDICTED: DNA mismatch repair protein MSH1, mitochondrial	7.43	-
		DNA mismatch repair protein MSH2	7.12	-
		PREDICTED: DNA mismatch repair protein MSH2	3.22	-
		PREDICTED: DNA mismatch repair protein MSH2	-	–9.62
	PMS1	DNA mismatch repair protein PMS1 isoform X1	-	–2.07
Cell wall related gene	expanin	PREDICTED: expansin-A4-like	9.81	-

		PREDICTED: expansin-A4	-	–8.23
	XTH	PREDICTED: probable xyloglucan endotransglucosylase/hydrolase protein 6	11.03	-
		PREDICTED: probable xyloglucan endotransglucosylase/hydrolase protein 28	2.18	-
		PREDICTED: probable xyloglucan endotransglucosylase/hydrolase protein 25, partial	-	–2.35
		PREDICTED: probable xyloglucan endotransglucosylase/hydrolase protein 23, partial	-	–2.75
		probable xyloglucan endotransglucosylase/hydrolase protein 28	-	–7.89
		probable xyloglucan endotransglucosylase/hydrolase protein 28	-	–9.59
Cellulose synthase	CS	PREDICTED: cellulose synthase A catalytic subunit 2	7.43	-
		PREDICTED: cellulose synthase-like protein E6 isoform X2	2.81	-
		PREDICTED: probable cellulose synthase A catalytic subunit 5	2.57	-
		Probable cellulose synthase A catalytic subunit 5	2.27	-
		Cellulose synthase-like protein E6 isoform X1	2.02	-
		Probable cellulose synthase A catalytic subunit 5	-	–2.68
		Cellulose synthase-like protein E6 isoform X2	-	–6.58
		PREDICTED: cellulose synthase-like protein E6	-	–8.15
		PREDICTED: cellulose synthase-like protein H1	-	–10.52
	Chitinase	PREDICTED: endochitinase A-like	7.43	-
		PREDICTED: acidic mammalian chitinase-like	-	–1.94

PREDICTED: chitinase domain-containing protein 1 isoform X1	-	–2.27
PREDICTED: acidic endochitinase	-	–2.33
PREDICTED: chitinase 2-like	-	–4.64
